# Microbial Interdomain Interactions Delineate the Disruptive Intestinal Homeostasis in Clostridioides difficile Infection

**DOI:** 10.1128/spectrum.00502-22

**Published:** 2022-09-26

**Authors:** Giovanny Herrera, Juan Camilo Arboleda, Juan E. Pérez-Jaramillo, Manuel Alfonso Patarroyo, Juan David Ramírez, Marina Muñoz

**Affiliations:** a Centro de Investigaciones en Microbiología y Biotecnología-UR (CIMBIUR), Facultad de Ciencias Naturales, Universidad del Rosario, Bogotá, Colombia; b Unidad de Bioprospección and Estudio de Microbiomas, Programa de Estudio y Control de Enfermedades Tropicales (PECET), Sede de Investigación Universitaria, Universidad de Antioquia, Medellín, Colombia; c Semillero de Investigación en Bioinformática-GenomeSeq, Seccional Oriente, Universidad de Antioquia, Medellín, Colombia; d Grupo de Fundamentos y Enseñanza de la Física y los Sistemas Dinámicos, Instituto de Biología, Facultad de Ciencias Exactas y Naturales, Universidad de Antioquia, Medellín, Colombia; e Molecular Biology and Immunology Department, Fundación Instituto de Inmunología de Colombia (FIDIC), Bogotá, Colombia; f Microbiology Department, Faculty of Medicine, Universidad Nacional de Colombia, Bogotá, Colombia; g Health Sciences Division, Universidad Santo Tomás, Bogotá, Colombia; h Molecular Microbiology Laboratory, Department of Pathology, Molecular and Cell-Based Medicine, Icahn School of Medicine at Mount Sinai, New York, New York, USA; Lerner Research Institute

**Keywords:** biomarkers, *Clostridioides difficile*, gut microbiome, interdomain interactions

## Abstract

Clostridioides difficile infection (CDI) creates an imbalance in the intestinal microbiota due to the interaction of the components making up this ecosystem, but little is known about the impact of this disease on other microbial members. This work has thus been aimed at evaluating the taxonomic composition, potential gene-associated functions, virulence factors, and antimicrobial resistance profiles of gut microbiomes. A total of 48 DNA samples obtained from patients with health care facility-acquired (HCFO) and community-onset (CO) diarrhea were distributed in the following four groups according to CDI status: HCFO/+ (*n* = 13), HCFO/– (*n* = 8), CO/+ (*n* = 13), and CO/– (*n* = 14). These samples were subjected to shotgun metagenomics sequencing. Although the CDI groups’ microbiota had microbiome alterations, the greatest imbalance was observed in the in the HCFO+/– groups, with an increase in common pathogens and phage populations, as well as a decrease in beneficial microorganisms that leads to a negative impact on some intestinal homeostasis-related metabolic processes. A reduction in the relative abundance of butyrate metabolism-associated genes was also detected in the HCFO groups (*P* < 0.01), with an increase in some virulence factors and antibiotic-resistance markers. A set of 51 differentially abundant species in the groups with potential association to CDI enabled its characterization, leading to their spatial separation by onset. Strong correlations between phages and some archaeal and bacterial phyla were identified. This highlighted the need to study the microbiota’s various components since their imbalance is multifactorial, with some pathogens contributing to a greater or lesser extent because of their interaction with the ecosystem they inhabit.

**IMPORTANCE** Clostridioides difficile infection represents a serious public health problem in different countries due to its high morbi-mortality and the high costs it represents for health care systems. Studies have shown the impact of this infection on intestinal microbiome homeostasis, mainly on bacterial populations. Our research provides evidence of the impact of CDI at both the compositional (bacteria, archaea, and viruses), and functional levels, allowing us to understand that the alterations of the microbiota occur systemically and are caused by multiple perturbations generated by different members of the microbiota as well as by some pathogens that take advantage of the imbalance to proliferate. Likewise, the 51 differentially abundant species in the study groups with potential association to CDI found in this study could help us envisage future treatments against this and other inflammatory diseases, improving future therapeutic options for patients.

## INTRODUCTION

Clostridioides difficile (a Gram-positive bacillus) is considered the main pathogen causing health care-associated infections in countries worldwide; 15% to 45% infection frequency has been described regarding community-acquired/onset and hospitalized patients, leading to more than 25,000 deaths annually and multimillion-dollar costs for health systems (1–5). Clostridioides difficile infection (CDI) can produce multiple alterations in the intestinal microbiota of patients suffering from it; patient state is aggravated by many factors, such as age, antibiotic use, and other comorbidities (6–11). Such alterations occur more frequently in an intrahospital setting where patients are exposed to many therapies associated with their delicate state of health, leading to an adverse effect on intestinal ecosystem equilibrium and thereby facilitating some pathogens’ growth and proliferation (11–13).

Recent studies have shown that *Faecalibacterium*, *Dorea*, and *Lachnospira* bacterial genera become reduced during CDI, as well as some prokaryotic archaea associated with protection against the disease (9). This has been accompanied by an increase in pathogens from the phylum *Pseudomonadota* (9–11, 13–15) and an increase in *Candida*, *Malassezia*, and *Blastocystis* (16–19). Such increase in pathogen populations creates suitable conditions for CDI maintenance and recurrence (7, 9, 13); this creates an ideal ecosystem for C. difficile development and proliferation due to a lack of commensal Pseudomonas able to produce short-chain fatty acids (SCFA) and secondary bile acids, leading to the exacerbation of symptoms and even death (13, 20).

Shotgun metagenomics sequencing, combined with other tools such metabolomics and metatranscriptomics, has enabled the detailed characterization of changes and relationships in the intestines of patients suffering inflammatory bowel diseases (IBD), such as Crohn’s disease (CD), ulcerative colitis (UC), irritable bowel syndrome (IBD), and colorectal cancer (CRC). Such an approach highlighting taxonomic, functional, and biochemical alterations has enabled the identification of biomarkers for such diseases’ diagnosis and treatment (21, 22). Most CDI studies have focused on delving into the taxonomic differences produced by C. difficile; this has led to some microorganisms being selected which have potential therapeutic use due to their protective role against CDI, as well as to exploring differences regarding fungal taxa abundance (10, 11, 23, 24).

However, studies concerning CDI-related intestinal microbiota disruption do not account for relationships among all the domains represented by a host’s wild intestinal ecosystem. This results in a lack of understanding about the complex processes associated with such disruption, highlighting the need for focusing on the study of microbiomes and considering a broader range of elements making up such ecosystems for improving our understanding of what happens regarding CDI.

This study used shotgun metagenomics for determining the composition of microbial communities (archaea, bacteria, and viruses), their functional profiles, and the relationships between the members of the microbiota and intestinal virulence- and antibiotic-resistance-associated molecular markers in patients suffering community-onset (CO) and HCFO CDI-associated diarrhea, compared to CDI-free diarrheal patients. Taxonomic composition profiles were found which agreed with those described in the pertinent literature, along with sets of characteristic differentially abundant species in the groups with potential association to CDI. Some metabolic processes’ functional profiling highlighted certain *Archaea* and *Faecalibacterium* species’ potential role in butyrate metabolism and oxidoreduction. Each group’s virulence and resistance profiles were determined; this led to increasing knowledge about the changes in microbial ecology potentially associated with CDI and improving a therapeutic approach to CDI patients.

## RESULTS

### The study groups presented differentially abundant bacterial and archaeal species.

Samples were grouped according to previously defined groups for highlighting differences in terms of taxonomic composition; an average of 16.4 million reads were obtained per sample (>33 Phred score). After eliminating host sequences, 15.9 million reads per sample were obtained, with the *Bacteria* domain being the most abundant (47% to 76%) (Table 1). The large number of unidentified sequences (no hits) in all groups (22% to 53%) was striking; there were more in the CO groups (Table 1). The similarities between HCFO groups are worth noting, as they had lower percentages of unidentified sequences and similar relative frequency for each taxonomic group found, characterized by a high percentage of bacteria.

**TABLE 1:** General statistics of taxonomical assignment of shotgun metagenomic reads

Group	Total reads	No hits (%)	Data for bacteria			Data for viruses			Data for archaea		
			%	Mean	SD	%	Mean	SD	%	Mean	SD
CO/–	197,797,349	51	48	6,792,810	3,571,867	0.07	10,232	12,420	0.04	5,463	3,813
CO/+	213,065,122	53	47	7,640,430	2,404,422	0.20	39,495	109,192	0.05	7,414	12,654
HCFO/–	139,362,722	22	76	13,183,284	3,249,136	2.00	291,200	756,346	0.02	3,209	5,041
HCFO/+	214,001,657	26	71	11,732,144	5,394,188	0.40	66,681	149,711	0.02	2,531	3,504

**FIG 1:**
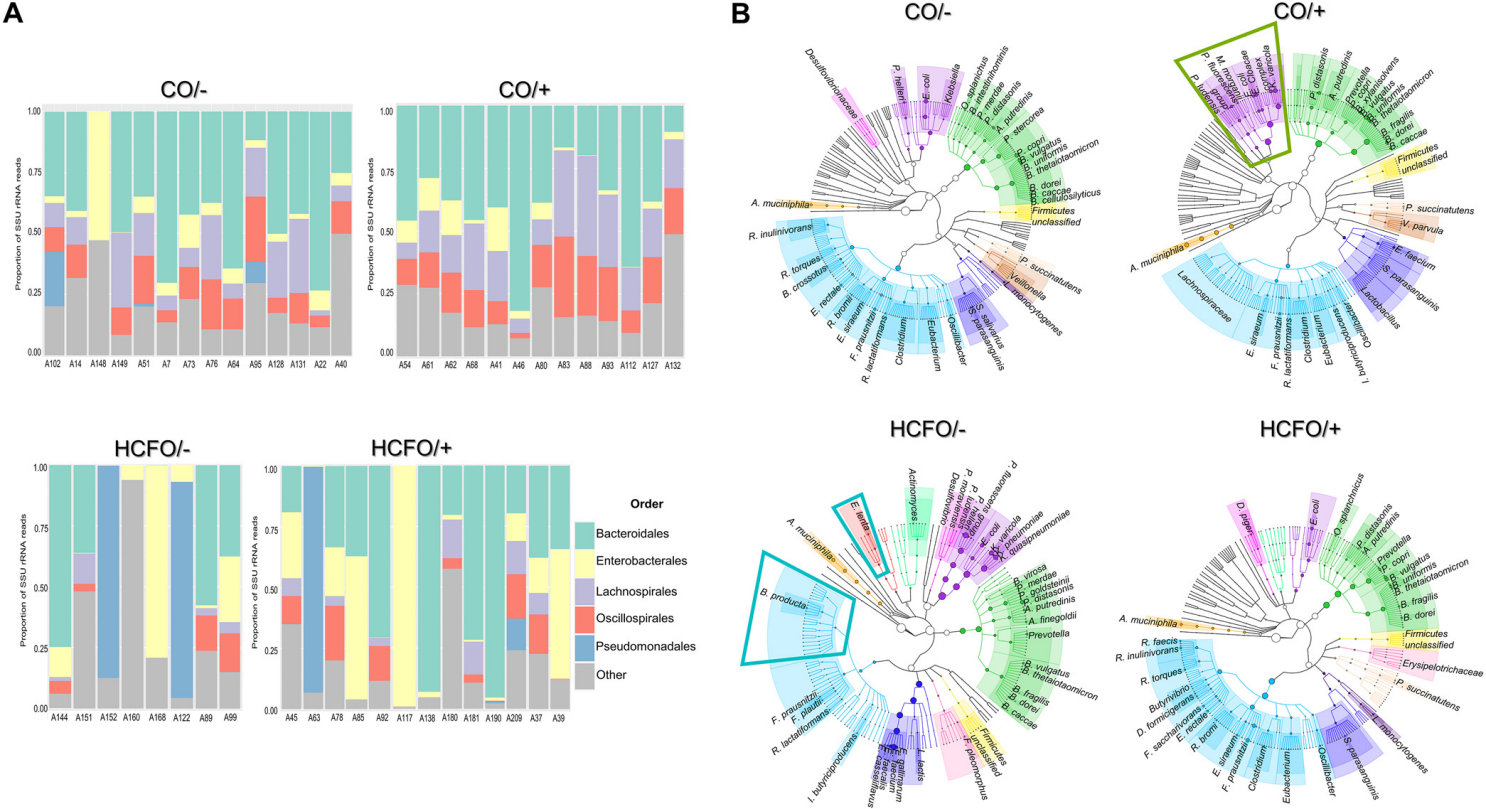
Taxonomic composition of HCFO and CO patients’ gut microbiome. (A) Reconstruction of taxonomic composition of bacterial orders following extraction of 16S gene reads using shotgun metagenomics data. (B) Dendrograms created using metagenomic phylogenetic analysis (MetaPhlAn) identifying differentially abundant bacterial species and showing characteristic species distribution in each group studied here. Colors were used for facilitating comparison of members from the same bacterial family in the study groups.

The bacterial population composition in each group described by 16S-rRNA marker reads had different profiles for each group. *Bacteroides*, *Lachnospira*, and *Oscillospira* dominated in the CO/– and CO/+ groups (Fig. 1A), while *Enterobacteriaceae* and Pseudomonas increased in the HCFO/ and HCFO/+ groups (Fig. 1A).

**FIG 2:**
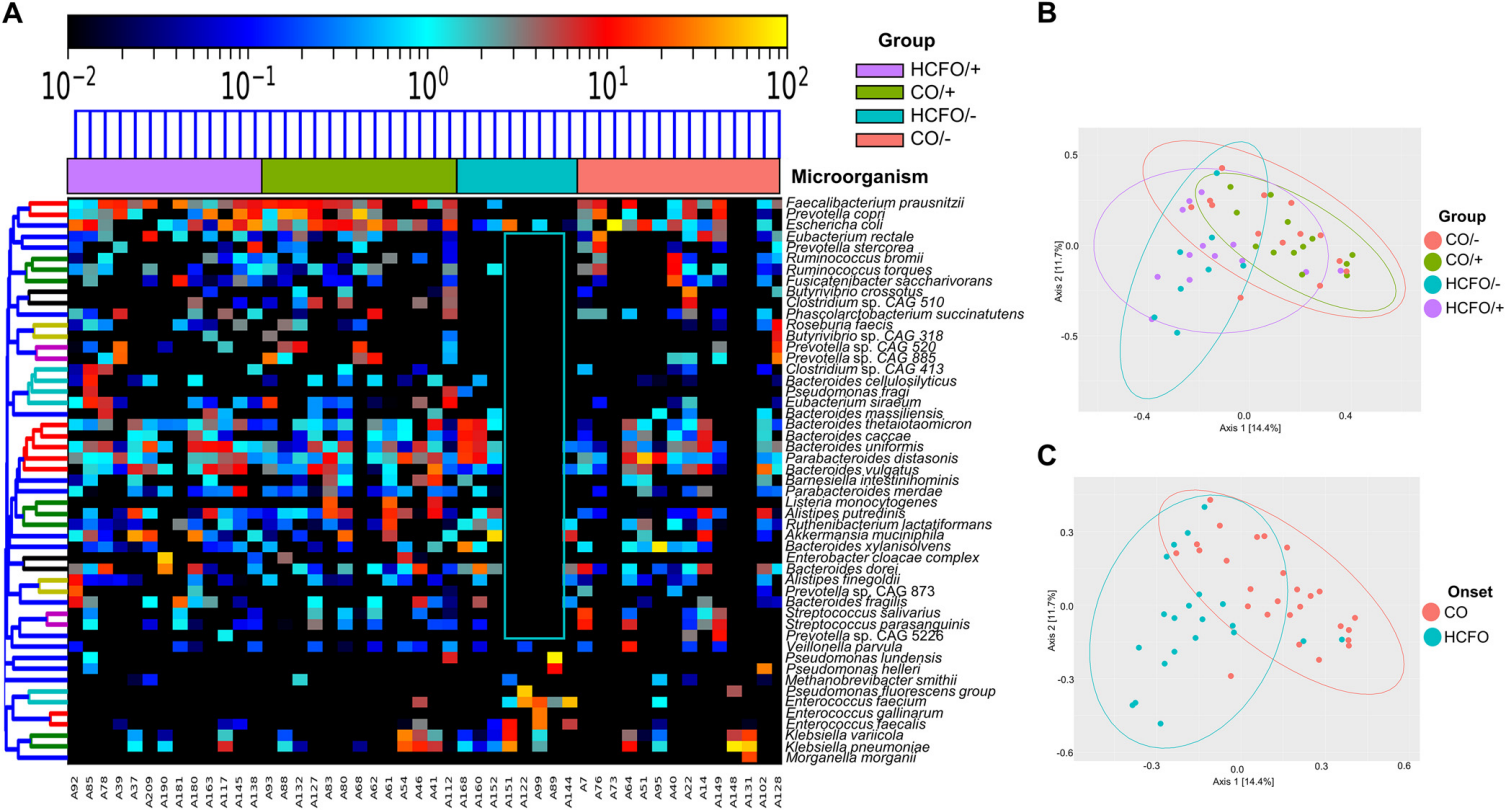
CDI and non-CDI diarrheic patients’ differentially abundant bacterial and archaeal species contribute to special differentiation. (A) Heatmap of 51 differentially abundant archaeal and bacterial species found in the study groups. (B) Principal-coordinate analysis (PCoA) based on the 51 differentially abundant species found in the study, showing the samples’ spatial separation for each group. (C) Principal-coordinate analysis (PCoA) showing sample separation according to onset.

The relative abundance of differentially abundant species identified by metagenomic sequencing had characteristic patterns (Fig. 2). For instance, we highlight a marked increase in common pathogens such as Klebsiella pneumoniae, Enterobacter cloacae, and Klebsiella variicola, along with an overall increase of *Pseudomonadota* phylum-related reads in the CO/+ group (Fig. 1B, green box). Few beneficial species accompanied by Eggerthella lenta were found in the HCFO/– group (Fig. 1B, blue boxes). The CO/– and CO/+ groups were characterized by greater diversity of members which have been associated with a beneficial profile; species from the *Bacteroidota* and *Bacillota* phyla were found, such as Odoribacter splanchnicus, Bacteroides uniformis, Roseburia faecis, and Roseburia inulinivorans (Fig. 1B). On the other hand, Akkermansia muciniphila was a species with a high relative abundance in all evaluated groups.

The analysis of differentially abundant species revealed 51 bacterial and archaeal species in the groups and the absence of up to 80% of the microorganisms described in 5/8 HCFO/– group samples (Fig. 2A, blue box). Differentially abundant species-based principal-coordinate analysis (PCoA) showed that both the HCFO (R^2^ = 0.12289, *P = *0.001) and CO groups (R^2^ = 0.07584, *P = *0.001) tended to cluster separately (Fig. 2B and C).

### External validation of differentially abundant species in the studied groups with potential association with CDI.

Analysis of compositions of microbiomes with bias correction (ANCOM-BC) allowed us to deepen into the species with a relative differential abundance in the CDI+ groups (HCFO/+ and CO/+) to determine which microorganisms had a potential association with the presence of C. difficile. The ANCOM-BC was performed on the study samples and displayed 48 species with a differential abundance. Some of these species had been previously described in the MetaPhlAn analysis (see Fig. S1 in the supplemental material). We carried out a validation of these differentially abundant species with a potential association with CDI by employing 27 publicly available samples belonging to two different studies, which we analyzed separately. Initially, the five samples belonging to the study of Milani et al. (25) reported less than 1 million reads per sample, whereas the 22 samples belonging to the study of Verma et al. (26) ranged from 23 to 34 million reads per sample. The ANCOM-BC performed on the data set of Milani et al. along with the negative samples of the present study indicated a total of 13 differentially abundant species. In contrast, the same analysis carried out on the data from Verma et al. along with the negative samples of our study yielded 52 differentially abundant species (Fig. S1). Clostridium clostridioforme was identified as the common differentially abundant species for the three data sets (Herrera [this study], Milani et al. [25], and Verma et al. [26]) in the samples positive for CDI. We found four different bacterial and archaeal species in Milani et al. and the other studies (two Milani and Herrera, two Milani and Verma), whereas we found six differentially abundant species herein and in the data set of Verma et al. (Fig. S1).

### Viral populations did not display differences between groups.

Viral communities accounted for 0.07% to 2% of all reads from the different groups, with the HCFO/– group having the highest percentage of these microorganisms (Table 1) (no statistically significant differences). Characteristic viral community profiles were observed in each group; IAS virus and *Faecalibacterium* phages predominated in the CO/– group, representing a third (33%) of this group’s viral populations, while the CO/+ group composition was characterized mainly by members of the Siphoviridae family (80% of the reads identified as virus), and *Bacteroides* phages were the most abundant (Fig. S2). There was an increase in Siphoviridae and Autographiviridae family members in the HCFO/– group (62% of viral sequences), accompanied by a relatively high abundance of Klebsiella phages, coinciding with the previously described differentially abundant species composition. Enterobacter phages such as those for Escherichia and *Enterococcus* dominated in the HCFO/+ group. However, such differences between viral families and species when comparing groups, onset, and CDI state were not statistically significant.

### Bacterial and viral populations depicted a strong correlation.

**FIG 3:**
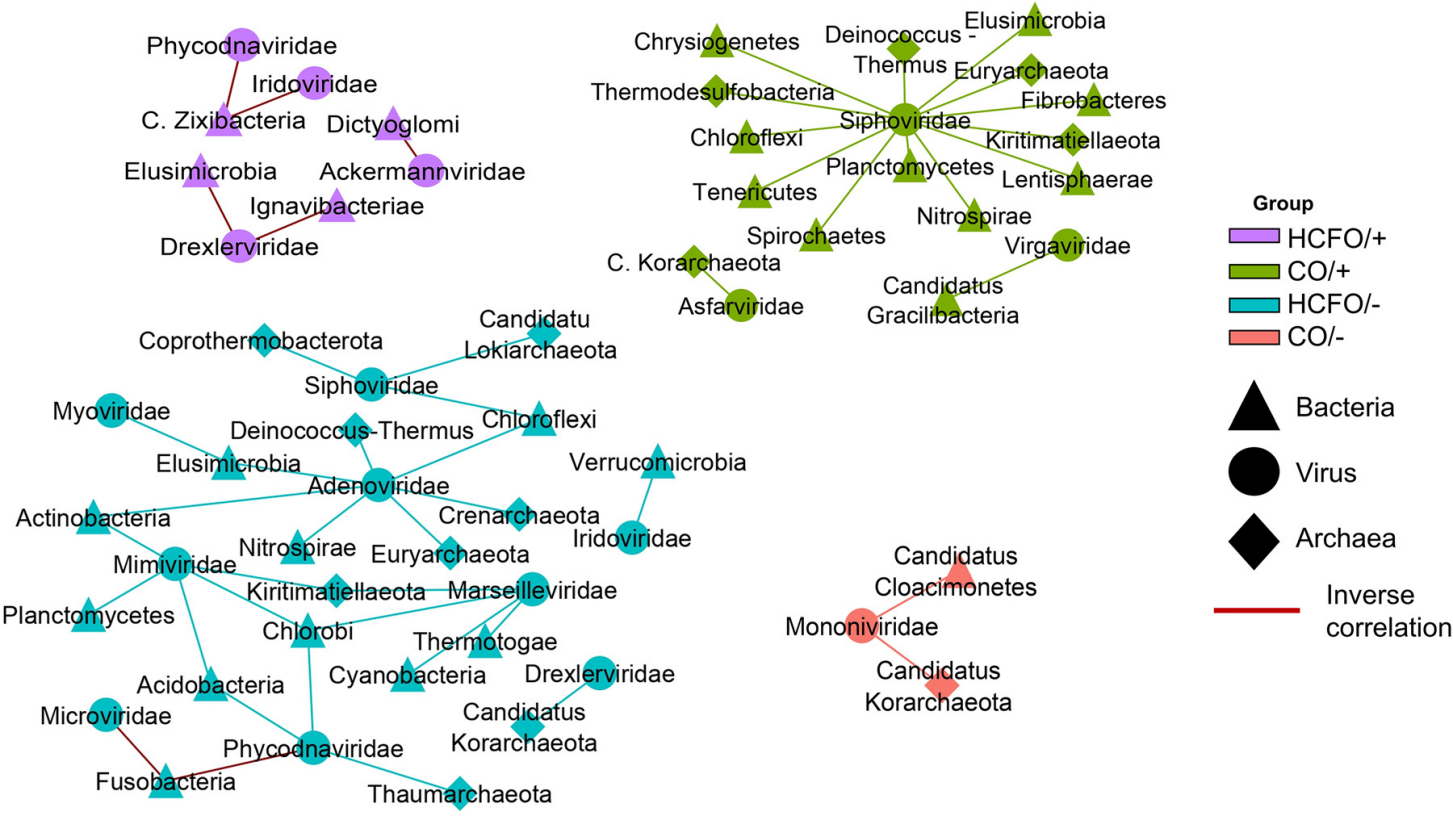
Positive and negative correlations between intestinal microbiota members from the patients being evaluated. Cooccurrence networks are shown for each study group with correlations higher than 0.75 and lower than −0.75.

Cooccurrence networks between viral families and archaeal and bacterial phyla revealed differences between groups (Fig. 3). Interestingly, the CO/– and HCFO/+ groups had fewer correlations, all being inverse (*ρ* <–0.75) in the group associated with intrahospital onset. For this type of onset, we observed an inverse proportional relationship between the abundances of some phages of enterobacteria, as well as other viruses with various bacterial families. The CO/+ group had numerous correlations, mainly between the Siphoviridae family and different bacterial and archaeal families, indicating the importance of this phage family and the wide range of hosts it can infect. Complex negative correlations were found in the HCFO/– group between viral families such as Picornaviridae and Microviridae with the same bacterial phylum such as Fusobacteria. A direct correlation between Siphoviridae and Adenoviridae with many archaeal and bacterial phyla was observed. Likewise, the complex correlations between the different families of *Archaea*, *Bacteria*, and viruses suggest an interaction between the domains, which may play a relevant role in the development of various diseases (Fig. 3).

### Metabolic pathways exhibited no differences between groups.

**FIG 4:**
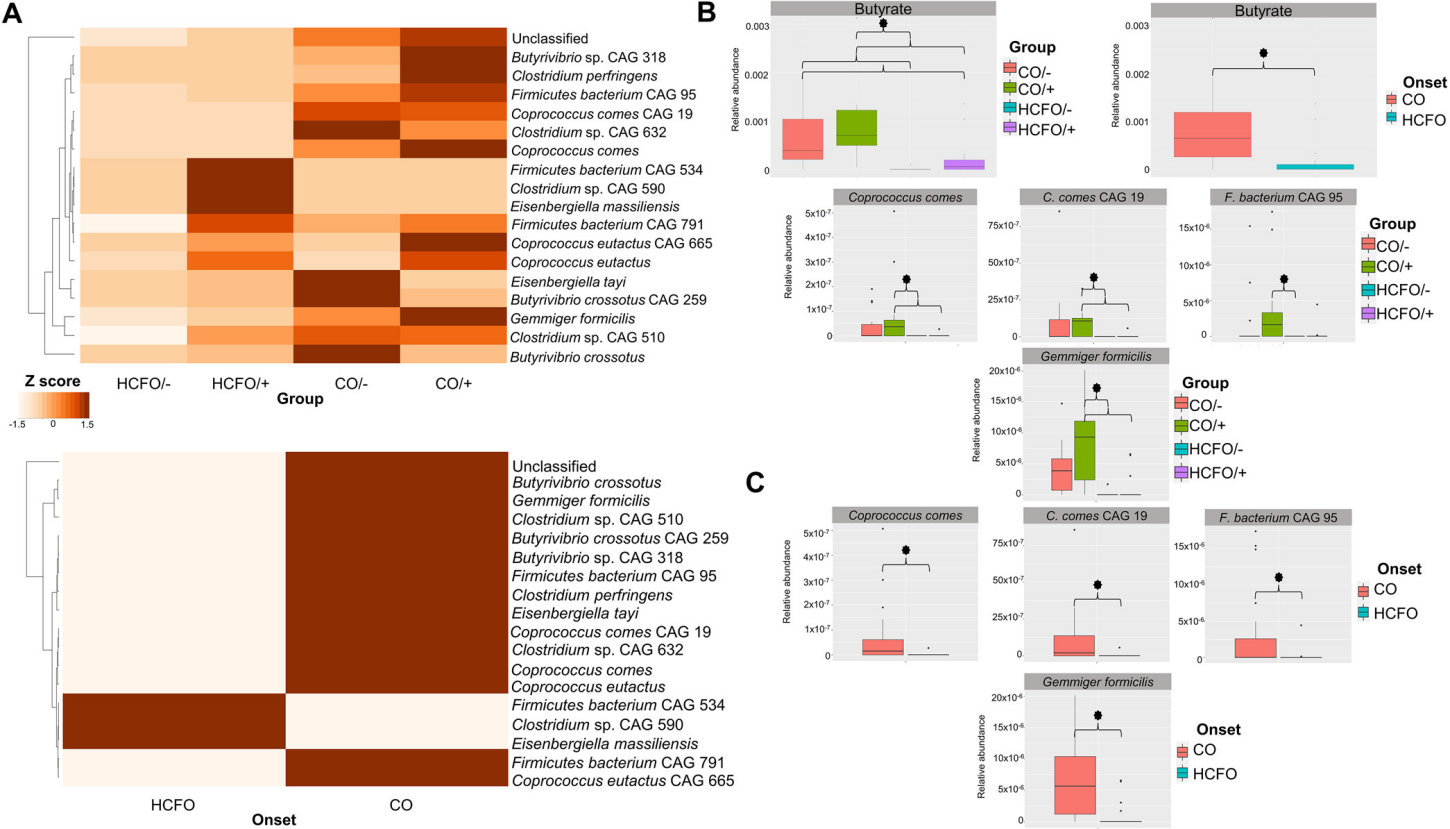
Loss of diversity regarding butyrate metabolism-associated bacteria in HCFO patients. (A) The contribution of each microorganism species in the samples by group and onset. (B) Comparison of the bacterial species contributing to butyrate metabolism in the study groups. (C) Box plots showing statistically significant differences between some species involved in butyrate metabolism by onset.

Multivariate analysis of the samples’ functional profiles revealed differences between the groups regarding the genes associated with 17 pathways; 5 were related to biological processes, and the other 12 were related to metabolic functions (Table 2). There were marked differences between the CDI-positive and -negative community groups compared to the HCFO/– group, as there were statistically significant differences concerning all the genes (Table 2). Analysis of butyrate metabolism proved interesting due to its potential impact on CDI’s natural history; there was an increase in bacteria contributing to such metabolic processes, mainly in the CO groups, accompanied by a reduction of all microorganisms potentially associated with butyrate metabolism in the HCFO/– group (Figure 4a; *P* values reported in Table 2).

**TABLE 2:** *P* values for multiple comparisons of potential gene-associated functions

Feature	Process	CO/– vs CO/+	CO/– vs HCFO/–	CO/+ vs HCFO/–	CO/– vs HCFO/+	CO/+ vs HCFO/+	HCFO/– vs HCFO/+
Butyrate metabolic process	Biological process	0.468	*0.002*	*0.000*	*0.040*	*0.008*	0.186
Cell wall assembly	Biological process	1.000	*0.001*	*0.000*	0.207	0.174	*0.022*
d-ribose catabolic process	Biological process	0.916	*0.002*	*0.002*	*0.007*	*0.008*	0.394
Regulation of apoptotic process	Biological process	0.425	*0.006*	*0.031*	0.120	0.346	0.152
Response to oxidative stress	Biological process	0.801	*0.001*	*0.001*	*0.013*	*0.010*	0.242
Acetone carboxylase activity	Metabolic function	0.452	*0.005*	*0.001*	0.476	0.174	*0.035*
Aryl-alcohol dehydrogenase (NAD+) activity	Metabolic function	0.956	*0.003*	*0.002*	*0.006*	*0.007*	0.417
Glucose-6-phosphate dehydrogenase activity	Metabolic function	0.600	*0.002*	*0.001*	0.215	0.100	*0.046*
Glutamate synthase (ferredoxin) activity	Metabolic function	0.682	*0.004*	*0.002*	*0.012*	*0.005*	0.437
Glycerophosphoinositol glycerophosphodiesterase activity	Metabolic function	0.997	*0.017*	*0.010*	0.062	0.051	0.331
Nonmembrane spanning protein tyrosine phosphatase activity	Metabolic function	0.952	*0.005*	*0.010*	0.189	0.222	0.122
Oligosaccharide reducing-end xylanase activity	Metabolic function	0.534	*0.006*	*0.002*	*0.022*	*0.005*	0.408
Oxidoreductase activity, acting on iron-sulfur proteins as donors	Metabolic function	0.989	*0.026*	*0.014*	0.068	0.054	0.384
Peptide-methionine (S)-S-oxide reductase activity	Metabolic function	0.501	*0.002*	*0.001*	0.482	0.206	*0.019*
Phosphatidylinositol-4-phosphate binding	Metabolic function	0.705	*0.002*	*0.004*	0.498	0.674	*0.013*
Tyrosine decarboxylase activity	Metabolic function	0.945	*0.045*	*0.030*	0.296	0.212	0.243
Uridylyltransferase activity	Metabolic function	0.484	*0.008*	*0.002*	*0.043*	*0.009*	0.336

Statistically significant differences were observed when comparing relative abundance between groups regarding genes and onset (Fig. 4B, Table 2). There were statistically significant differences regarding the microorganisms involved in such metabolic processes between groups and onset, i.e., *Coprococcus comes* (Kruskal-Wallis chi-squared value = 15.477, *P = *0.001451; W = 422, *P = *0.0004825), *Flavobacteria bacterium* (Kruskal-Wallis chi-squared value = 7.8338, *P = *0.04957; W = 366.5, *P* = 0.02415), and Gemmiger formicilis (Kruskal-Wallis chi-squared value = 17.658, *P = *0.0005173; W = 460.5, *P = *0.00008817) (Fig. 4B and C).

Differences were found regarding the metabolic process associated with oxidoreductase activity concerning the abundance of genes associated with such processes between groups (Kruskal-Wallis chi-squared value = 12.542, *P = *0.005739) and onset (W = 446.5, *P = *0.0007032) (Fig. S3A). Statistically significant differences were found regarding the contribution of Faecalibacterium prausnitzii to this process in the HCFO groups (Kruskal-Wallis chi-squared value = 14.22, *P = *0.00262) (Fig. S3B).

### C. difficile infection-positive groups had increased virulence factors.

**FIG 5:**
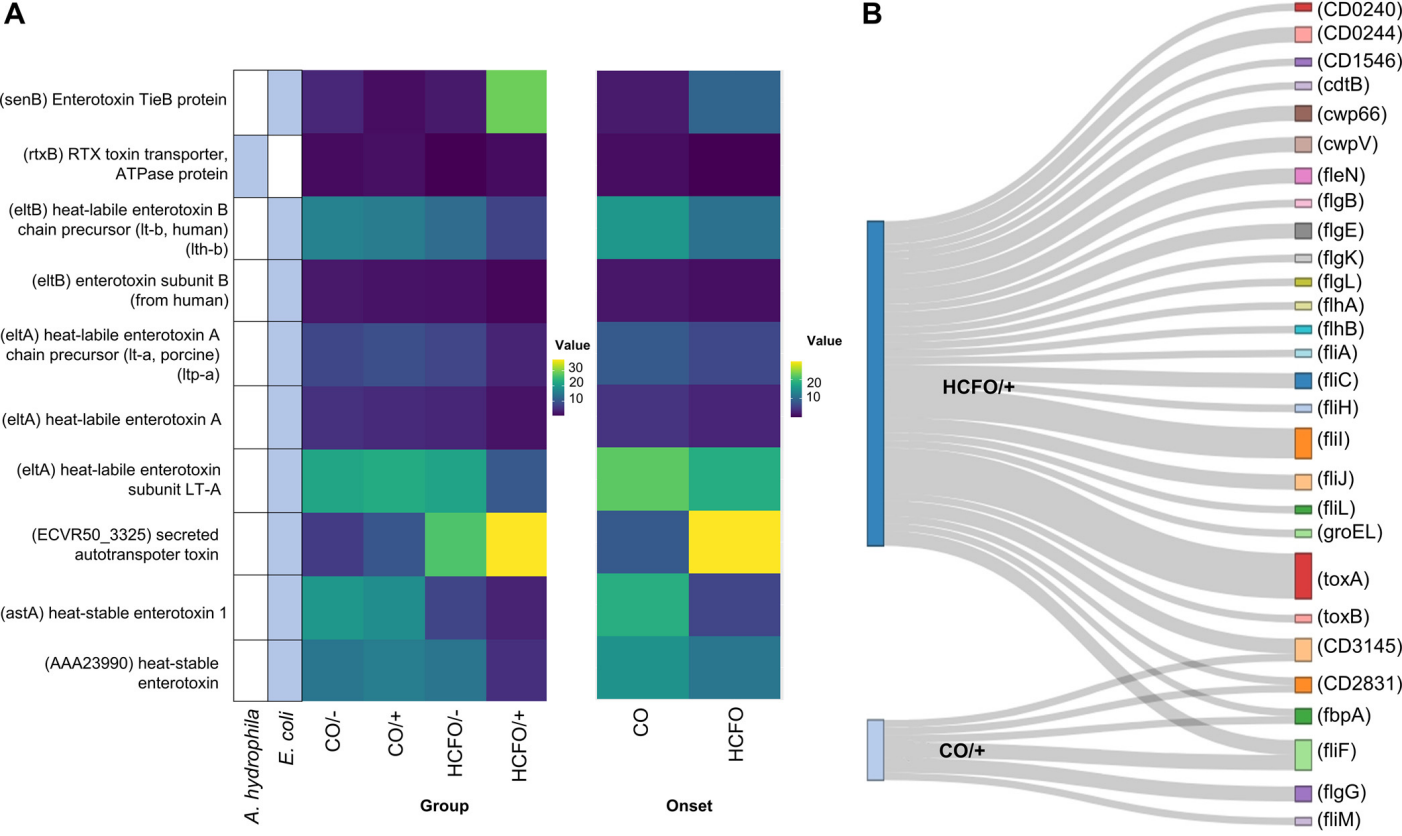
Toxins and virulence factors are more related to HCFO groups. (A) Heatmap of the toxins and associated microorganisms found in each group. (B) Distribution of C. difficile*-*specific virulence factors found in the groups positive for such microorganisms.

Analysis of virulence factors (placing special emphasis on toxins) revealed an increase in toxin-related genes in CDI-positive groups, especially intrahospital-related ones (Fig. 5A), with the Escherichia coli toxins (*astA*) heat-stable enterotoxin 1 (W = 405, *P = *0.003821) and (*rtxB*) RTX toxin transporter, and ATPase protein (W = 378.5, *P = *0.03206) being different. C. difficile-encoding virulence factors, which were only found in the HCFO/+ and CO/+ groups, were analyzed (Fig. 5B). It was noted that the HCFO/+ group had the largest number of these virulence factors, including toxin A- and B-related genes which were not found in the CO/+ group.

### The HCFO groups presented multiple antibiotic resistance genes compared to the other groups.

**FIG 6:**
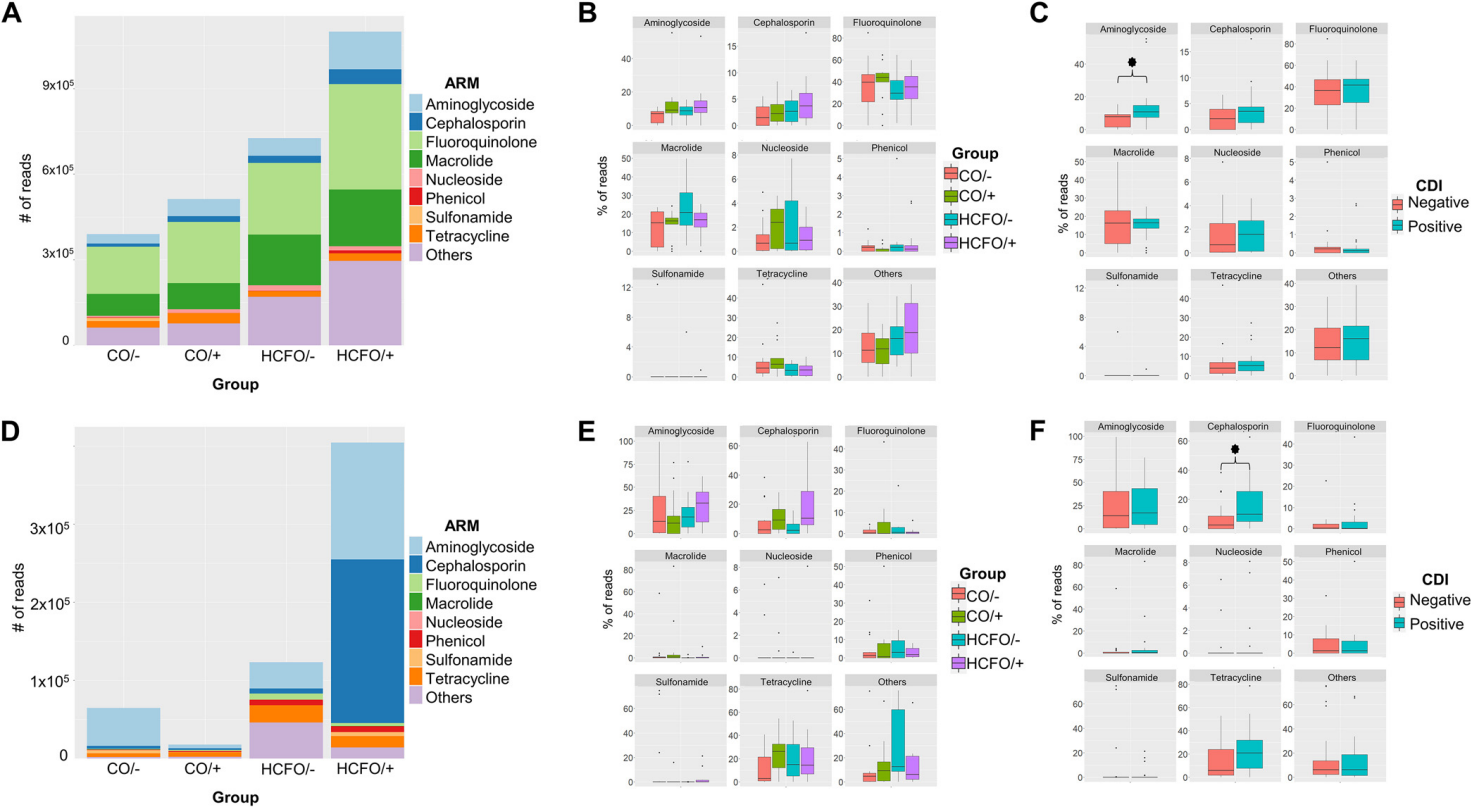
Increased genomic and plasmid ARMs in HCFO groups. (A) Distribution of genomic ARMs regarding antibiotic class per study group. (B and C) Boxplots of each antibiotic involved in the ARMs found in groups and by onset. (D) Distribution of plasmid ARM reads regarding antibiotic class by groups. (E and F) Boxplots of each antibiotic involved in plasmid ARMs found by group and onset.

Genomic and plasmid analysis of antibiotic-resistant genes revealed that the HCFO groups had more antibiotic resistance marker (ARM) reads, especially in the HCFO/+ group (not statistically significant) (Fig. 6). Analyzing ARM genomic composition (Fig. 6A) revealed that most markers were fluoroquinolone-resistant ARMs; however, there were no statistically significant differences regarding any of the ARMs analyzed, while statistically significant differences were observed when grouping samples according to CDI state between the percentages of ARM-encoding genes associated with aminoglycoside resistance (*P = *0.0096), with CDI-positive groups having the largest number of these markers.

Plasmid ARMs were mostly cephalosporin- and aminoglycoside-resistant markers (Fig. 6D). An increase in the relative abundance of cephalosporin-specific ARMs was observed in the CDI-positive patient group (*P = *0.0224). Differences were consistent with genomic findings, as no statistically significant differences were observed regarding the other ARMs (Fig. 6E and F).

## DISCUSSION

The study groups’ bacterial taxonomic composition (Fig. 1A) agreed with the information obtained by previous deep sequencing of a single 16S rRNA gene marker in a set of samples which included those analyzed in this study (9). Analysis of some 16S-rRNA hypervariable regions enabled precise characterization of the bacterial populations and accounted for C. difficile’s negative impact on beneficial bacterial populations (Fig. 1). This impact has been observed in many studies (10, 14, 15, 25, 27); it has been described as altered microbiota regarding ecosystem balance, having reduced *Bacteroidota* and *Bacillota* phyla (11, 13), probably due to the administration of antibiotics, thereby producing an increase in inflammatory processes facilitating the proliferation of pathogenic bacteria from the *Pseudomonadota* phylum. This would sustain patients’ adverse conditions, leading to a recurrence of CDI and making them susceptible to other infections (11).

The changes between taxonomic groups in this study could show that the CDI-related microbiota imbalance arises from the relationships between intestinal ecosystem elements, i.e., not being modulated by an isolated member. Evidence of this is the impact (even at the metabolic level) of relationships established between different beneficial markers, such as *Odoribacter*, *Faecalibacterium*, and *Roseburia* in community-associated groups. (Fig. 1B and Fig. S3); this is related to a positive influence on intestinal ecosystem homeostasis, bearing in mind the ability to produce SCFA (i.e., butyrate), which has been associated with triggering inhibitory signals concerning the expression of proinflammatory cytokine transcription factors creating an environment with low inflammation levels (28–33).

A reduction in the amount of these beneficial microorganisms has an impact on intestinal microbiota; this is taken advantage of by common pathogens such as Pseudomonas, *Morganella*, Klebsiella, and *Enterococcus* (as observed in the intrahospital groups: Fig. 1B), which have been associated with inflammatory states and the worsening of patients’ states in other studies, thereby hampering their clinical and therapeutic management (34–37). The presence of other microorganisms such as Eggerthella lenta in the HCFO/– group (Fig. 1B) suggested a negative effect on intestinal microbiota, which has been associated with inflammatory diseases such as colitis and other complications such as bacteremia, even though its mechanisms of pathogenicity are poorly understood (38–41).

The observed profiles of the differentially abundant species with potential association with CDI between the data sets may be due to clinical and sociodemographic factors. Due to the absence of clinical data from our samples, we could not establish comparisons at this level with the other data sets. Thus, it is relevant to highlight that the absence of the factors here and the technical differences (sequence length and amount) represent the principal limitations of this investigation. Therefore, there is a need to deepen both the results obtained here and future comparisons based on clinical and population data. Consequently, this information will allow a more in-depth evaluation of the potential associations between CDI and microorganisms that contribute to the imbalance of the intestinal ecosystem, which occurs in CDI and other inflammatory pathologies. Moreover, the impact of individual and temporal variations on the intestinal microbiota composition (42) hinders the extrapolation of the results to studies carried out in diverse populations such as European and American ones. For this reason, it is fundamental to increase the data at a regional scale to obtain more accurate comparisons that lead to promising results in the management and treatment of CDI.

Despite the lack of differences between the groups’ viral communities’ taxonomic composition, the cooccurrence networks indicated direct correlations in most groups (Fig. 3); this could have been related to the viral lysogenic cycle, suggesting provirus-related phage populations and that their increase resulted from an increase in *Bacteria* and *Archaea* populations which they infected. Recent reports suggest that this could have arisen from a reduction in available nutrients due to phage ability to obtain information from inside a host cell regarding the metabolic activity of the bacterial populations they infect, i.e., for determining whether such conditions might promote phage proliferation (43, 44).

Similarly, a model of interaction between phage P22 and Salmonella enterica serovar Typhimurium led to identifying subpopulations which were provisionally resistant to phage infection, enabling phage production without leading to a reduction of host populations (45). However, further studies are required for demonstrating the impact of such relationships, since little is known about the switch between lytic and lysogenic cycles in the intestinal microbiota.

The reduction of butyrate metabolism-associated genes found in this study, mainly in intrahospital groups (Fig. 4), was an extremely relevant finding, as this metabolite contributes to intestinal homeostasis regarding immune and inflammatory response modulation, intestinal barrier formation, and maintenance of colonocyte energy metabolism (28, 46). Such a reduction might be related to a deterioration in HCFO patients’ condition compared to that of CO patients; this highlights the importance of controlling the intestinal microbiota balance for patients’ gradual improvement.

The broad variety of microorganisms associated with butyrate metabolism found in all groups studied (Fig. 4) could have resulted from a broad group of commensal and pathogenic bacteria’s ability to produce this metabolite from different substrates (47). The forgoing is very important due to butyrate’s many benefits regarding intestinal homeostasis and lipid and carbohydrate metabolism (46, 48–50), meaning that it must be maintained within the intestinal ecosystem for promoting microbiota equilibrium.

Virulence factor analysis revealed an increase in the genes encoding Escherichia coli-associated toxins in the HCFO/– group, mainly the secreted autotransporter toxin (Sat) (Fig. 5A) inducing cell damage during enteroaggregative infection by this microorganism (51), which could trigger complications for patients in this group. It is worth stressing the increase in sequences identified as C. difficile virulence factors in the HCFO/+ group compared to the CO/+ group (Fig. 5B). The HCFO/+ group had a higher degree of microbiota imbalance, which would have provided suitable environmental conditions for pathogenic microorganism proliferation and the transfer of genes playing an important role regarding health (52). This would support the hypothesis that the presence of C. difficile along with the imbalance caused in the microbiota produced by an increase in virulence factors leads to a worsening of patients’ health-related conditions.

It is also worth noting that antibiotic administration could contribute to eliminating bacterial populations; this would create disturbances in their equilibrium due to an impact on many members’ diversity and abundance, in turn contributing to the development of resistance to antibiotics among members of the microbiota by acquiring genes from the environment and other bacteria (53), representing a threat to public health. Factors which could be related to determined ARMs must thus be identified, as in this study the ARMs where identified in HCFO group (Fig. 6); however, future studies are needed to identify the factors that could be related to its presence in this population in developing countries as Colombia. The available works that have analyzed antimicrobial resistance in HCFO have provided an association between the environment of the patients and the multiple treatments to which they are subjected due to the diseases they suffer from (54, 55). Chromosome and plasmid resistance markers’ differential patterns (Fig. 6) reveal the imbalance in these patients’ intestinal microbiota generated by many factors, such as the presence of C. difficile, which could contribute to the transfer of resistance genes among microorganisms, thereby worsening patients’ clinical condition, limiting their treatment, and even placing their lives at risk.

This has thus been the first metagenomics study regarding the setting for C. difficile-associated diarrheic patients in Colombia. The results suggested that individual microbial members do not cause microbiota imbalance but, rather, that microbial ecology (the relationships established with other individuals and their environment [56]) plays an essential role, and thus any imbalance affects microbial communities’ composition to different extents, including a possible metabolic impact and thus an impact on patients’ health.

Further studies are required for determining the impact on the expression of the genes found here. Pharmacological surveillance of antibiotic treatment in the general population must be strengthened, as this could be triggering an increase in different microorganisms’ resistance. These results should contribute to identifying pathogenic microorganism’s characteristic of the imbalance produced by CDI and potentially beneficial ones that could counteract the infection’s impact and which, therefore, might be candidates for probiotics; however, future research must be aimed at verifying differentially abundant species’ roles regarding health and establishing these microorganisms’ intestinal ecosystem homeostasis.

## MATERIALS AND METHODS

### DNA selection and shotgun metagenomic sequencing.

A total of 48 DNA samples stored in the Universidad de Rosario’s Centro de Investigaciones en Microbiología and Biotecnología (CIMBIUR) cryobank from a 2019 study by Muñoz et al., (57) were selected for this work. The samples had been classified into four groups according to the state of CDI and where the infection had been acquired, following Society for Healthcare Epidemiology of America and Infectious Diseases Society of America guidelines (58) as described in Muñoz et al., (59): community onset positive for CDI (CO/+, *n* = 13), community onset negative for CDI (CO/–, *n* = 14), health care facility-acquired positive for CDI (HCFO/+, *n* = 13), and health care facility-acquired negative for CDI (HCFO/–, *n* = 8). The samples forming the groups were randomly selected in line with the following technical requirements: amount of DNA, purity, and available volume. Metagenomics sequencing was used for the selected samples (Illumina platform, Paired-end (PE)150 Q30, >80%; 4G raw data/sample) at Novogene (Sacramento, CA, USA).

### Evaluating data quality and filtering.

FastQC (60) and MultiQC searches (61) were made of the data for ascertaining read quality; the Trimmomatic read-trimming tool for Illumina next-generation sequencing (NGS) data (62) was used for trimming low-quality sequences (Q score, <20) and those with less than 150-bp size. Bowtie 2 (63) was used for the decontamination step when aligning reads from human host sequences with the human genome reported in NCBI (Genome Reference Consortium Human Build 38 [GRCh38], accession number PRJNA31257).

### Taxonomic binning and profiling and identification of differentially abundant species by group.

Two approaches were used for describing the composition of the communities in the samples. The phyloFlash pipeline (64) was used for specifically describing the samples’ bacterial and archaeal communities; this involved extracting 16S rRNA gene sequences. The Kraken (65) tool for assigning taxonomic labels to short DNA sequences was used for the samples’ taxonomic binning. The gplots (66) programming tool was used for producing heatmaps; differences were evaluated by Kruskal-Wallis test and *post hoc* analysis using Dunn’s test with Benjamini-Hochberg stepwise correction (67), with the 0.05 significance level in Rstudio software (68).

MetaPhlAn 3.0 software (69) was used for profiling the composition of microbial communities; GraPhlAn (70) was used for creating the graphics. The Kruskal-Wallis test and *post hoc* analysis were used for evaluating differences regarding differentially abundant species—abundance between groups. The phyloseq package (71) was used for importing, storing, analyzing, and graphically displaying already clustered phylogenetic sequencing data, along with beta diversity using the differentially abundant species found by a principal-coordinate graph based on Bray-Curtis dissimilarity. Permutational multivariate analysis of variance (PERMANOVA) was used for evaluating centroid differences, i.e., adonis (analysis and partitioning sums of squares using dissimilarities) and vegan functions (descriptive community ecology-related statistics package) (72).

### External validation of CDI-associated species.

We performed an analysis of compositions of microbiomes with bias correction (ANCOM-BC) to validate the bacterial and archaeal species that were differentially abundant in the CDI-positive groups. The ANCOM-BC is a robust analysis that controls the false-discovery rate (FDR) and presents a statistical approach that allows evaluating the reproducibility and reducing the bias associated with differences in sampling (73). For this analysis, we simultaneously compared the groups: CO/– and HCFO/– versus CO/+ and HCFO/+, considering a significance level of 5%.

The differentially abundant species associated with the CDI-positive groups observed here were compared to previously published data of Milani et al. (*n* = 5) (25) and Verma et al. (*n* = 22) (26). These two studies used shotgun metagenomics on their samples, where CDI-positive patients presented diarrheal symptoms similar to those of the present study. CDI-negative samples of these two studies were not considered, as they were patients without diarrhea symptoms; thus, they did not meet the inclusion criteria of our research.

For the validation, we retrieved the raw data belonging to CDI-positive samples from the Sequence Read Archive (SRA) and submitted it individually to the previously described preprocessing (quality control, filter and trimming, and decontamination). Subsequently, a taxonomic assignation was performed using MetaPhlAn, as described previously. Finally, each data set was compared to the CDI-negative samples of our study to determine the differentially abundant species present in the CDI-positive samples of each study. For this, we applied the ANCOM-BC with a significance level of 5%.

### Bacteria and virus cooccurrence network.

Spearman’s nonparametric rank-order correlation with Benjamini-Hochberg correction was used for corelating viral families and archaeal and bacterial phyla, taking *P < *0.05 values as being significant and strong correlations (*ρ* < −0.75 and *ρ* > 0.75) (R package psych). Correlations were then graphed in the Cytoscape 3.9.0 network visualization tool, data integration, and analysis software (R packages igraph, ggraph, and Rcy3).

### Functional profiling.

Humman3 was used for metabolic pathway functional profiling and reconstruction (69), using MaAsLin 2.0 (74) (Rstudio) for evaluating differences between groups by multivariate analysis; *P < *0.05 values were taken as being significant. A Kruskal-Wallis test was used when association was identified, along with a Dunn test with Benjamini-Hochberg correction for *post hoc* analysis using multiple comparisons (*P < *0.05 for significant associations).

### Identifying virulence factors.

The Basic Local Alignment Search Tool (BLAST) (75) was used for identifying virulence factors by aligning the decontaminated reads obtained with Bowtie 2 against reads from the virulence factor database (VFDB) (http://www.mgc.ac.cn/VFs/) (released 17 June 2021) (76). Results were given in terms of the highest percentage of identity, using a 95% cutoff point.

### Identifying antibiotic resistance markers.

The Comprehensive Antibiotic Resistance Database (CARD, version 3.1.3, released 5 July 2021) (77) Resistance Gene Identifier (RGI) tool was used for evaluating and predicting antibiotic-resistance markers and analyzing metagenomic reads. The Kruskal-Wallis test was used for evaluating the differences between marker reads by type of antibiotic used in the groups, along with *post hoc* analysis by Dunn test with Benjamini-Hochberg adjustment for multiple comparisons (*P < *0.05 significance).

### Ethics approval and consent to participate.

The current project was conducted with the approval of the Universidad del Rosario’s Research Ethics Committee (approval number 339). This study was considered low risk according to Colombian Ministry of Health Resolution 8430/1993.

### Data availability.

The data are publicly available at the European Nucleotide Archive (ENA) repository under accession number PRJEB50313.
