# Supplementary material for: Microbial Interdomain Interactions Delineate the Disruptive Intestinal Homeostasis in Clostridioides difficile Infection
Source: Microbiol Spectr. 2022 Sep 26;10(5):e00502-22. doi: 10.1128/spectrum.00502-22 (PMC9602525; doi:10.1128/spectrum.00502-22)
Supplement: Supplemental file 1 — Fig. S1 to S3. Download spectrum.00502-22-s0001.pdf, PDF file, 0.5 MB [file spectrum.00502-22-s0001.pdf]

Supplementary material

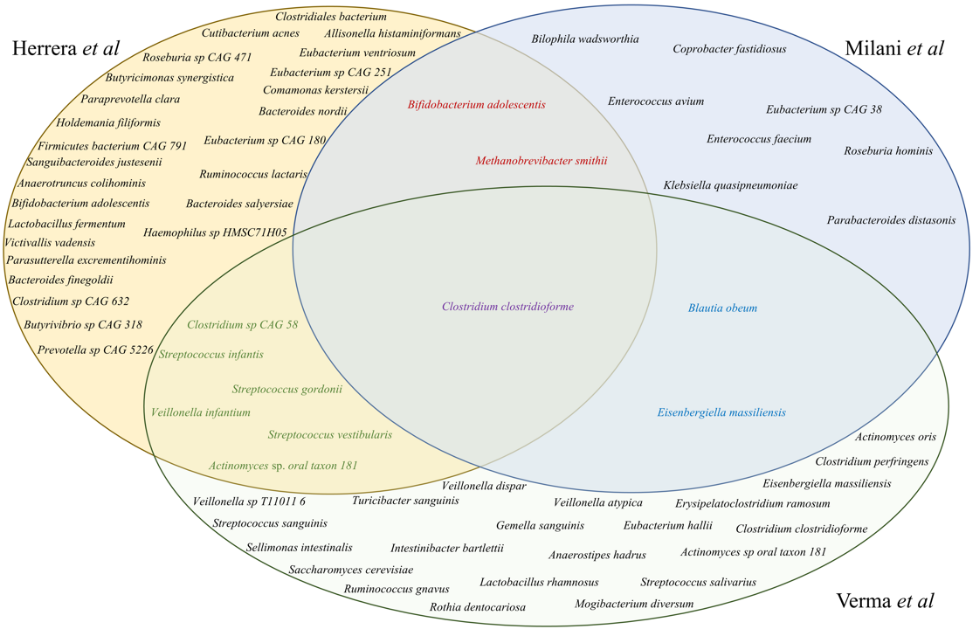

Figure S1. Differentially abundant species with potential association to CDI and its external validation. Venn diagram of the differentially abundant species in the CDI-positive samples compared to the negative samples from the present study and the two independent public datasets.

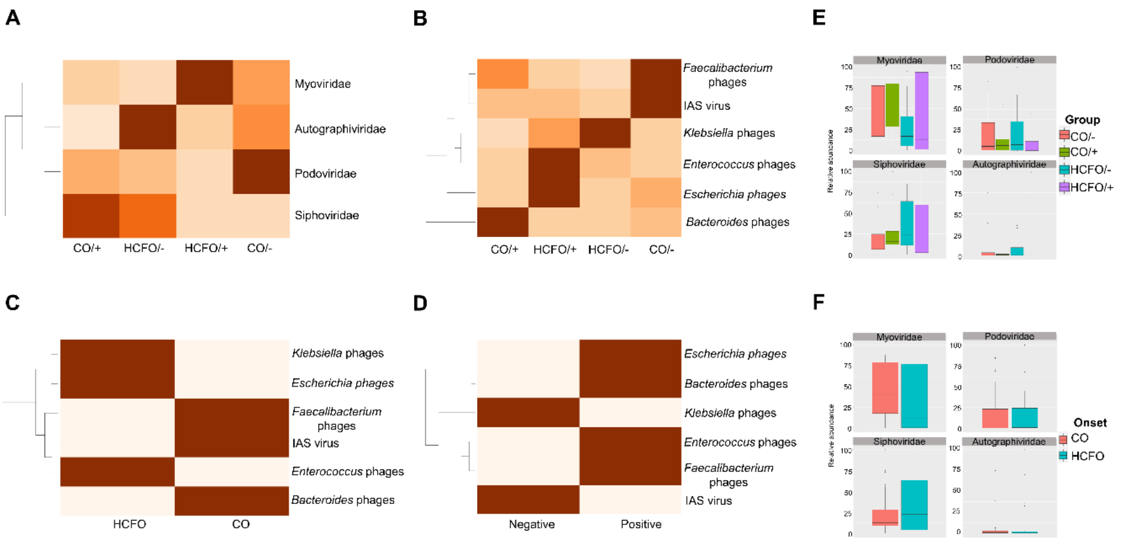

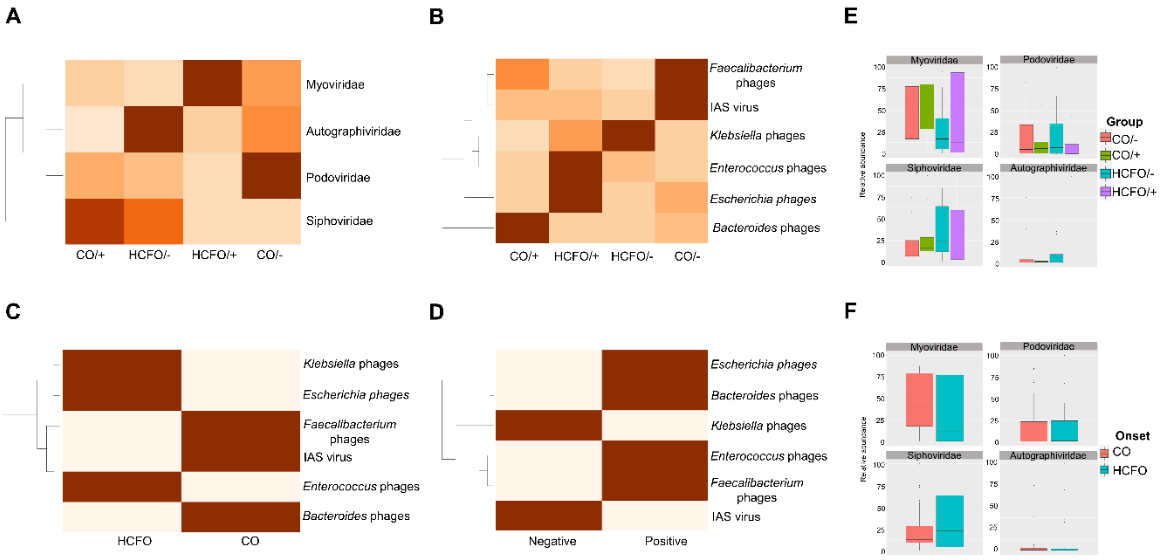

Figure S2. Viral populations were related to bacterial composition. Distribution of the main phage families and species found in the study groups. A) Heatmap of viral families by group; B) Heatmap of phage species by group. C) Heatmap of viral families by onset; D) Heatmap of phage species by onset. E and F) Box plot showing absence of differences between viral families by group and by onset.

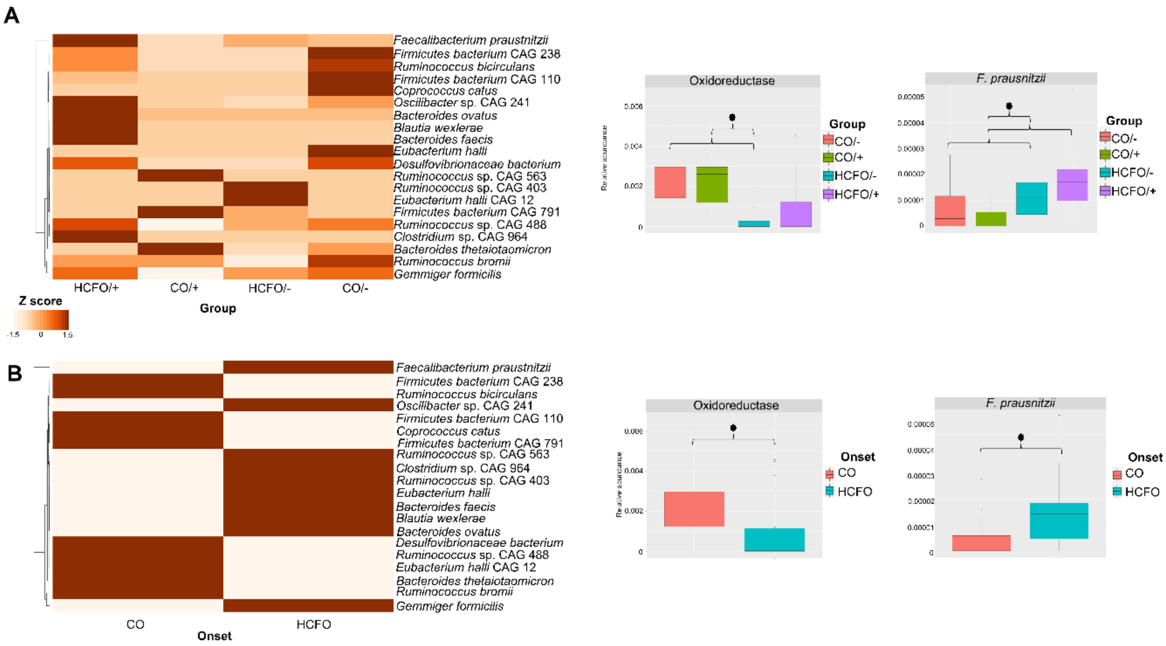

Figure S3. The role of *F. prausnitzii* in oxidoreductase metabolism. A) Heatmap of the microorganisms involved in oxidoreduction by group, along with their box plots showing statistically significant differences; B) Heatmap of the microorganisms involved in oxidoreduction by onset, accompanied by box plots showing statistically significant differences.
